# Supplementary material for: Suicidal behavior risks during adolescent pregnancy in a low-resource setting: A qualitative study
Source: PLoS One. 2020 Jul 22;15(7):e0236269. doi: 10.1371/journal.pone.0236269 (PMC7375578; doi:10.1371/journal.pone.0236269)
Supplement: S1 Data — (DOC) [file pone.0236269.s001.doc]

**M: I want us to start our today’s discussions, and the first question is this; have you been experiencing depression for a period exceeding two weeks? Feeling stressed and having a lot of depression?**

R: No. 1 – No.

**M: Someone else?**

R: Silence.

**M: Have been experiencing depression for a period exceeding two weeks?**

R: No. 7 – No.

**M: Silence**

R: No. 5 – No.

**M: Silence**

R: No. 2 – No.

**M: Silence**

R: No. 3 – No.

**M: Silence**

R: No. 4 – No.

**M: Silence**

R: No. 6 – No.

**M: Ok, the second question is asking; have you lost interest on things that you liked doing; things that you used to do in the past but now you are no longer interested in doing them? Interest; have you lost interest for a period exceeding two weeks? Lets say things that you were interested in doing like going to the church, attending meetings etc. is there something that you are no longer interested in; that you used to be interested in; in the past?**

R: No. 6 – No.

**M: Silence**

R: No. 5 – I used to be interested in walking a lot; but now I don’t feel as active as I used to.

**M: Ok,**

R: No. 4 – No.

**M: No.?**

R: No. 5.

**M: No. 5 – you have said that lately you are no longer interested in some things that you used to be interested in. what are these things? That you are no longer interested in?**

R: I used to like walking around; but now I don’t feel so anymore. I simply stay at home.

**M: How long has this being?**

R: Ever since my pregnancy was three months old.

**M: Ok, anyone else? Things that you used to be interested in but now you are no longer interested in them?**

R: No. 3 – No.

**M: Silence**

R: No. 2 – No.

**M: Silence**

R: No. 1 – No.

**M: Silence**

R: No. 4 – No.

**M: Ok, someone else?**

R: Silence.

**M: I understand you go through very difficult times often. And so I want to know; what tough things have you been experiencing in your mind? Things that have been bringing you stress?**

R: Silence

**M: We have agreed to open up, right?**

R: (chorus answer) Yes.

**M: Let’s start. We can begin from there. What things have been very difficult for you; stressing you? Let’s begin with you. Ok, pass it over.**

R: Everything is going on well with me. I don’t have a problem.

**M: Yes,**

R: No. 5 sorry, No. 4 – I don’t seem to be doing well at times. Like at night when I sleep; my body temperatures rise to the extend that I can not sleep, some other times I sit up and I feel my stomach is aching too much, you find that I don’t have peace.

**M: Has that one made you to think so much / be stressed up?**

R: No. 4 – Stress.

**M: For how long?**

R: No. 4 – let’s say for instance now; after about three weeks is when I started experiencing that when I was diagnosed to be pregnant. Up to date, I have never felt comfortable.

**M: Is there anything else that has been in your mind causing you stress?**

R: No. 4 – No.

**M: Someone else?**

R: No. 3 – There isn’t.

**M: Silence**

R: No. 2 – There isn’t

**M: Silence**

R: No. 1 – there isn’t

**M: We have agreed that you open up, right?**

R: (Chorus answer) Yes,

**M: Ok,**

R: No. 7 – There isn’t.

**M: What has been stressing you? Let us share. Something that has been in your mind bothering you for a long time?**

R: No. 6 – In the past I would feed well but now I no longer eat as before. When it comes to sleeping, I used to sleep deeply but now whenever I sleep, I keep waking up often over and again and not be able to get sleep again. In walking, I never used to like walking but now I walk.

**M: What is making you lack sleep at night?**

R: No. 6 – Some other times when I don’t get sleep at night, I sit up. I don’t even sleep.

**M: What makes you lack the sleep?**

R: No. 6 – Sorry?

**M: What is causing the problem?**

R: No. 6 – Ever since I got pregnant, and in fact the first pregnancy I miscarried,

**M: Yes,**

R: No. 6 – When I miscarried ever since then; After the miscarriage is when these problems began up to now when I got this second pregnancy. You find that I am not getting sleep, I am stressed so much, and I think so much to the extend that I don’t get sleep. When I wake up, when I loose sleep even if it’s at midnight I don’t sleep again till morning.

**M: For how long has this lasted?**

R: No. 6 – Since 2017.

**M: Have you been examined by a professional like a Medical Service Provider or a Community Health Volunteer to assist you?**

R: No. 6 – Yes I shared with my sister and she counseled me. Whenever I share with her she counsels me and I start improving. Whenever she counsels me, I find myself doing much better.

**M: Someone else who has been experiencing stress? Thinking too much?**

R: No. 5 – I am not stressed.

**M: Silence**

R: No. 4 – I am not stressed.

**M: The next question we are asking; how are you doing in your day to day programme in your relations or where you are engaged in? Would you say you are doing well or there is a variance?**

R: No. 4 – Ever since I was diagnosed pregnant, I am occasionally upset. Whenever I see people I feel upset. If a child comes my way I feel like beating it up, yes.

**M: Ok, how are you doing in your relations at home with your parents, your closest friends, are you relating normally or there is a change? Pass it over,**

R: No. 2 – I am doing well, normally

**M: Someone else?**

R: Silence

**M: How can you describe your relations with your parents? Your family relations or your closest friends? Pass it over to her,**

R: No. 6 – Is good.

**M: Yes,**

R: No. 3 – There is no problem at all.

**M: How can you describe your relations with your friends or family? Including your closest friends?**

R: No. 1 – Is good.

**M: Silence**

R: No. 5 – Is good.

**M: Yes, and have you ever contemplated terminating your life? Thinking of terminating your life?**

R: No. 5 - No. I have never contemplated.

**M: Silence**

R: No. 4 – No.

**M: No. 4 you have said No?**

R: No. 4 – Yes.

**M: Silence**

R: No. 2 – No.

**M: Silence**

R: No. 1 – No.

**M: Silence**

R: No. 7 – No.

**M: Silence**

R: No. 6 – Yes, I contemplated terminating my life, about two times. I wanted to terminate my life when I got my first pregnancy. I was not relating well with parents, I was not having peace. I attempted about two times, to terminate my life. The second time I got pregnant, I have never had peace.

**M: What have you said causes you the desire to contemplate terminating your life?**

R: No. 6 – Whenever I carry a pregnancy and also not relating well with parents. I don’t find peace; I want to terminate my life.

**M: What makes you not relate well with your parents?**

R: No. 6 – You find that they don’t want to see me in my pregnancy,

**M: Yeah,**

R: No. 6 – We are not relating and not even talking with each other.

**M: And during those occasions, what did you use to try to terminate your life?**

R: No. 6 – Because I was not having peace, I wasn’t having peace to enable me stay alive.

**M: Did you use anything to try to terminate your life?**

R: No. 6 – I used a rope.

**M: How were you finally helped? Since that time?**

R: No. 6 – A TBA counseled me. She counseled me and I got courage to move on.

**M: Ok, I now want to ask you another question and especially during this time of your pregnancy. Where do you seek services from, when you are pregnant? Is it from a TBA or the hospital or where? We can begin from there. No, we can even begin with you. What is your number?**

R: No. 6 – Hospital.

**M: No.?**

R: No. 6

**M: Yes,**

R: I receive services from the hospital and at other times from the TBA.

**M: You have said that you receive services from both?**

R: Yes.

**M: And, someone else?**

R: No. 7 – I receive services from the church and from home.

**M: How about on issues to do with your pregnancy? Where do you seek services from? Is it from the TBA or from the Professional Healthcare Service Provider?**

R: No. 7 – Hospital.

**M: And, (pass it over)**

R: No. 5 - From the parents, TBA and the hospital.

**M: No?**

R: No. 5

**M: Silence**

R: No. 4 – From the parents, TBA and the hospital.

**M: Silence**

R: No. 3 – From the Professional Healthcare Service Provider

**M: Silence**

R: No. 2 – From the parents, hospital and the TBA.

**M: Silence**

R: No. 1 – From the parents and the TBA.

**M: Ok, and who directs you on where to seek services from? Who influences where you seek services from? Who tells you where to seek for services from? Let’s begin here.**

R: No. 1 - Parents

**M: Yes,**

R: No. 2 – the parent and the TBA.

**M: Yes,**

R: No. 3 – My husband and my mother.

**M: Yes,**

R: No. 4 – My husband

**M: Yes,**

R: No. 5 – My husband and parents.

**M: Yes,**

R: No. 7 - From parents and the TBA.

**M: Yes,**

R: No. 6 – From parents

**M: Ok, and so we have shared; some have shared that they get services from the TBA, others from the hospital, actually from the TBA and the hospital. And I want us to understand; do you find these service providers adequately prepared to offer you these services? Are they skilled enough to offer you the services you require? Let’s begin there.**

R: No. 7 – Is able to offer the required services.

**M: And this is in regard to mental health issues like stress. So, you said you sought for services where? And you say your number.**

R: No. 6 – From my parents and the TBA.

**M: So were they able to assist you stopping your problem of wanting to terminate your life?**

R: No. 6 – Yes, they were able to assist me.

**M: Ok, how did they assist you?**

R: No. 6 – Offering me counseling, simply counseling during this time of pregnancy.

**M: You had informed us that you sought for services from a TBA?**

R: No. 6 – Yes.

**M: So what services did you receive from a TBA?**

R: No. 6 – Offering me counseling

**M: Yes,**

R: No. 6 – Yes, during this period of my pregnancy.

**M: Yes, No? You had informed us that you seek for services from the hospital and from a TBA. The question I want to ask you is this; do you find your Professional Healthcare Service Provider or the TBA adequately equipped to handle your mental healthcare issues like stress?**

R: Yes, they assist me.

**M: On the side of TBA, what services do you receive?**

R: On the side of TBAs, when I happen to invite her to my home she comes, offers me the services I require and counseling to a pregnant person.

**M: Ok, what kind of counseling to a pregnant person?**

R: Talks on loving your husband more, loving others too and praying a lot.

**M: How about on the side of the hospital? What services do you receive there?**

R: When I visit the hospital, I benefit with clinic services and they offer me counseling as I go home.

**M: What else still in the same question? You said you benefit with services from both sides? What services do you receive from a TBA?**

R: On the side of TBA, whenever she visits me in my home she counsels me, this is my first time to be pregnant; I have never been pregnant before. I have been to so much stress and a feeling of not been comfortable. So she tells me to persevere a time will come and all this will be over. When I come to visit the Professional Healthcare Service Provider, I come for clinics; he counsels me on what a pregnant person should conduct herself, how to relate with the husband and such like things. The same with my parents; they offer me counseling. They tell me that we should live happily and in harmony.

**M: Do you find a TBA capable of handling your mental health issues like stress? In the community?**

R: No. 3 – Yes.

**M: Yes,**

R: No. 3 – Whenever I visit the Professional Healthcare Service Provider, I am offered good counseling and informed on the things that I require using as a pregnant person.

**M: No. 2. Right? You had shared that you receive services from both ends; from the TBA and from the Professional Healthcare Service Provider. Let’s begin on the side of TBA. How do you benefit?**

R: No. 2 – When I visit a TBA,

**M: Yes,**

R: No. 2 – She offers me counseling on how I am supposed to conduct myself during my pregnancy, as this is my first pregnancy. She tells me how the pregnancy should be, both to my relations and to my parents. In the hospital, when I attend clinic; we are usually counseled and the Professional Healthcare Service Provider informs me as well, how I should be prepared.

**M: How does a TBA assist you on issues of mental health or stress related?**

R: She tells me that I should not stress myself and I should not view the challenges at my home as a big issue to the extend of contemplating terminating my life. She gives me courage.

**M: How about at the hospital? How does the Professional Healthcare Service Provider assist on issues of mental health?**

R: I am told not stress myself with such like thoughts and be strong.

**M: Ok, let’s come back here. What would you propose be done to your Professional Healthcare Service Provider or your TBA so that they can offer better mental health services to you? What would you like to be done? Let’s begin here. What do you think can improve greatly the performance of your service provider on issues of mental health? Let’s say for instance the Professional Medical Healthcare Service Provider or the TBA? Let’s begin with you.**

R: I would like the TBA to be God fearing.

**M: Yes,**

R: And be a role model in the community; such that whatever she is telling me, I have seen her has done it herself and has helped so many people with it. The other thing that I would like to see in a TBA is keeping secrets. Whatever I share with her, she should keep it as a secret between me and her. She should not tell everyone.

**M: Do you see them doing that? Do they keep secrets?**

R: Yes, the one we have keeps secrets. You never get to hear anything talked anywhere about her or about yourself.

**M: What would like to see most in your mental health service provider improved? Issues of stress etc**

R: No. 6 - Simply praying for her to keep it up; in whatever she is doing. She should avoid sharing people’s secrets. This TBA is good in the community. She is well contented and not a problem to us.

**M: Ok, I want to ask those amongst you who said were married; Does a TBA assist you when you are involved in family conflicts? Do you find the TBA in a position to offer help?**

R: Ask the question again.

**M: Number?**

R: Silence

**M: In the community,**

R: Yes,

**M: Do you find the TBA being of any help when it comes to family conflicts? Like conflicts between you and your husband or conflicts involving yourself and the parents to your husband? Can a TBA come in to offer help?**

R: I usually see her come in; in some situations, in some homes but I have not got to that level personally of having conflicts with my parents and my husband to the extend that we would require her assistance. We disagree the same way people do and we make up again.

**M: But you have said to some cases they assist? Do you think they help in conflict resolution?**

R: They do help. This is especially for these young girls who are getting pregnant while in school. She goes to the extend of talking to the parents of the girls, to the extend that the parents now agree to accept the girls as they are.

**M: What is your experience in the community? Are TBAs able to assist in family conflicts? Conflicts involving yourself with your husband or with your mother in law?**

R: For instance when she pays me a visit to my home, sometimes / sometimes let’s says for instance; there are some pregnancies that show up with varying attitudes like wanting to hate people at times. There is a time I had conflicts with my parents and not my husband. And when I called the TBA, she told me that there are some pregnancies that are chaotic and others are not. And so she advised me. She told me if I reach a level where I am likely to disagree with the parents I simply keep quiet or forward the issue to my husband. She advised me not to be exchanging words with my parents. She helped me. She told me my response should be to keep quiet.

**M: Wow! Is there anyone else who was assisted by a TBA? Pass it to her to share with us.**

R: No. 6 – When a TBA pays me a home visit, she assists me. She tells me to persevere, if it’s a case of disagreeing with parents, it happens and I should not keep on exchanging words with them. This is because keeping on exchanging words with them will add on to a lot of stress. I should simply zip up. If she appears to be initiating a talk that might lead to conflicts, I should simply keep quiet. She told me to practice staying up in silence throughout.

**M: Lets come here, No. 3; in your community, have you heard of an incidence where a TBA was able to help resolve family conflicts?**

R: No. 3 – Personally I don’t want to lie. I have not yet come into contact with a TBA. I am still young in marriage and starting to know these things.

**M: Is a TBA able to detect or know when one is stressed? For those that have been close to a TBA, is she able to know when one is stressed? Yes, pick up and explain to us your case;**

R: Yes, a TBA is able to…. (Intervened)

**M: No.?**

R: No. 5 – a TBA is able to know when one is experiencing stress. This is because when many times you call her to check on your pancreas, you hear her ask you; ‘could you be thinking too much?’ pancreas problem is sometimes associated with stress. When you go or you happen to meet with her and you tell her that you are having an headache, she is able to detect that you are stressed and she is able to offer you counseling.

**M: This side, you seem to be too quiet? Is a TBA able to detect stress in a person? Start by saying your number;**

R: Silence

**M: In your own opinion; is a TBA able to detect that you are suffering from stress? Or you have mental disorders? This side you are so quiet?**

R: Silence.

**M: Ok, I want you to share with us about how the TBA came to know that you were pregnant or who was the first person to know that you are pregnant? When you got pregnant?**

R: No. 1 – She was informed by my parent.

**M: Ok,**

R: No. 2 - She was informed by my parent.

**M: Number?**

R: No. 2

**M: Yes,**

R: No. 4 – I stayed for a month. When I didn’t receive my monthly periods I came to visit the Medical Healthcare Service Provider. I have since been visiting every single month without skipping. So I came for the tests, and I was confirmed pregnant.

**M: How about the TBA? How did she get to know that you were pregnant so that you could start benefiting with her services?**

R: No. 4 – After I was confirmed pregnant, I now informed the TBA.

**M: Between the TBA and your parents; who was the first person to know that you were pregnant?**

R: No. 4 - My parents.

**M: Ok.**

R: Yeah,

**M: How did the TBA learn that you were pregnant?**

R: No. 5 – The TBA learnt that I was pregnant because when I got pregnant, I didn’t inform anyone including my own husband. So after about three weeks I started having problems with the pancreas. I didn’t know that I was pregnant and so I called the TBA to come and help me out with the pancreas problem. When she touched me, she told me that I was pregnant and I should not be checked by any other TBA. If I need help, I should be calling her specifically.

**M: Ok, number?**

R: No. 6 -

**M: How did the TBA get to know that you were pregnant? And who was the first person to know that you were pregnant?**

R: No. 6 – I was the first person to know that I was pregnant after I didn’t receive my monthly periods. I stayed for one month, then another month came and went, and it was during the second month that I confirmed that I was pregnant. I started feeling like I wanted to vomit, I didn’t have a liking for anything, and so I went to the TBA. It’s the TBA who was the first person to know that I was pregnant. So it was the TBA who first knew that I was pregnant.

**M: Now I want you to tell me; looking back to your communities, is a TBA skilled to handle stress related issues? You had shared but we can talk it further. Mental disorders; is a TBA skilled to handle and treat them?**

R: To anyone or to the sick person?

**M: In relation to the pregnant women.**

R: Silence

**M: We had shared this but we can talk about it deeper.**

R: No. 5 – A TBA is able to help one overcome stress.

**M: In which way?**

R: No. 5 – through offering counseling and telling her what a pregnant woman undergoes during her pregnancy.

**M: You are so quiet here. I want us to turn and answer this question; what prevents / can prevent a TBA from giving you mental health treatment? In your community? What challenge do you see? Such that you can see this one is an hindrance? To treatment of stress and other mental health related issues? What prevents them from giving you that support?**

R: No. 5 - Sometimes when one is thinking that they have a lot of stress, they have so many things occupying their mind, and they can share that one with the TBA who intern doesn’t keep it secret! So in such a case, this pregnant woman will keep her problems to herself.

**M: Yeah, the question is; what challenges are there preventing you from getting help to mental health issues?**

R: Silence

**M: What prevents you from getting helped for mental health issues in your community?**

R: Silence

**M: Lets open up and share so that we can wide up.**

R: Silence.

**M: Does it mean when you are stressed you are able to get help?**

R: Silence.

**M: What could be hindering you from getting the necessary support from stress and thinking too much?**

R: Silence

**M: Who wants to answer that one?**

R: Silence

**M: What can hinder you from getting the help you require for mental health issues?**

R: No. 5 – You may be ready for marriage, and you get married to a home that keeps secrets! A home where the family members are don’t cares and they don’t care about your concerns. And so you need courage to be able to share those concerns with someone outside the family circles to someone else. This is because you are usually afraid! You don’t want to share with outsiders and finally the family members get to hear about it.

**M: Now I want each one of you now to answer me this question; what are your views on involving TBAs in mental health treatment? Involvement of TBAs in mental health treatment? What are your views?**

R: Silence

**M: Can TBAs be involved in mental health treatment?**

R: No. 1 – Yes,

**M: Why? Why do you say yes?**

R: Silence

**M: Ok, pass to number two. Pass to number two to tell us.**

R: Silence

**M: Number? Begin by telling us your number.**

R: Silence

**M: Should TBAs be involved in mental health treatment?**

R: No. 2 – Yes they should be.

**M: Yes,**

R: No. 2 - Why because when you are having challenges,

**M: Yeah,**

R: No. 2 – You can share with your parent, who is near to you,

**M: Yeah,**

R: No. 2 - And if your TBA was not within reach,

**M: Yeah,**

R: No. 2 - Is telephoned and comes and explains to you the reasons for the problems that you are facing like stress and helps you reduce the stress levels that you have.

**M: Ok, so you have said that a TBA is able to help you lower your stress levels, did you share with us number two? How is she able to lower your stress?**

R: No. 2 – You share your experience with her, and she gives you comfort.

**M: Should a TBA be informed in mental health treatment? In your community?**

R: No. 4 - When a TBA is…. (Intervened)

**M: Number?**

R: No. 4

**M: Yes,**

R: No. 4 - When a TBA comes home,

**M: Yeah,**

R: No. 4 – She comes like sometimes let’s say; you know many parents simply play hide and seek. They hide, they hide and they are simply keeping it secret. But the moment I call a TBA, you know I open up to her,

**M: Yeah,**

R: No. 4 – I tell her that this is what I am experiencing and she offers me counseling. The other day I called her because whenever I ate food, I would have a problem with my pancreas. I would always feel uncomfortable. Even if I see what or what! I simply feel not wanting to be associated with it. And you would find that mostly it’s craving for lemons! So when I came and asked her why I had a craving for lemons, she told me that lemons are bad! They contain a lot of acid and are the ones making my pancreas to pain. And so you would find that whenever I asked my parents, I would simply laugh it out and encourage me to persevere because it’s the way of life and so I told her that I was going to call a TBA to come and help me out because she wasn’t willing to tell me and the TBA will not be afraid to tell me.

**M: So your personal opinion is that TBAs should be involved in mental health treatment?**

R: No. 4 – Yes.

**M: Why do you think they should be involved? Why should they be involved? Why should we involve them in mental health treatment?**

R: No. 4 – Lets say for instance whenever I have a problem, I call her.

**M: Yes,**

R: No. 4 - And is able to assist me where I am unable to.

**M: Yes,**

R: A TBA should be involved in …… (Intervened)

**M: Number?**

R: No. 5 – A TBA should be involved in mental health treatment because mostly when one is pregnant and they tend to get stressed, they are able to / the baby is the one who tends to get more stressed than the mother. The baby will be burdened by those thoughts and she will have a lot of problems. Again a TBA should be involved in issues of mental health because all the problems and challenges facing a pregnant woman are familiar to the TBA and they are usually willing to offer help.

**M: Yes,**

R: No. 6 – A TBA should be involved in many of these issues because and especially to these pregnant women. This is because it might happen that today you are having problems and you have no one back home to support you. So you telephone someone to go and bring you a TBA. In such cases, a TBA helps very much in assisting women, thus they should be involved in some of these issues.

**M: Yes, we are specific on mental health issues. Why do you think a TBA should be involved?**

R: No. 6 – is because she assists a lot.

**M: She assists in which way?**

R: No. 6 – By giving you courage to persevere so that she can offer counseling to you the pregnant woman so that you don’t get so much stressed.

**M: I want us now to talk about a common problem that is very popular amongst people. This is the problem of thinking about; what exactly makes women / young pregnant women (your age mates) contemplate terminating their lives? What mainly makes them think about terminating their lives? You are so quiet, let us begin there.**

R: Silence.

**M: Do we all know what can make us (young pregnant girls) contemplate terminating our lives? In this community?**

R: Silence

**M: Let’s actively participate we finish up.**

R: No. 4 – Lets say some of these things; some of these things back at our homes we disagree with our parents, and so you sit down and think so much about it; and so you decide; ‘instead of staying alive on this earth, I would rather terminate my life so that they live in peace’. So there are other days when one disagrees with my husband. Some people will disagree with their husbands; especially if the husband is a drunkard or even where others are not even drunkards at all. Personally I have not had conflicts with my husband. For those that are drunkards, one comes and starts beating her from nowhere. In the process of being battered, the pregnant woman considers the act and concludes; now, even before I was married I was not in good relations with my parents, and I don’t have good relations with my biological parents! Here comes, she is married and is not in good relations with the in laws and the husband so she decides instead of being a burden to my parents and my husband, I would rather terminate my life.

**M: Had you said your number?**

R: No. 4

**M: Ok, someone else?**

R: No. 5 – when these young girls get pregnant, they usually have so many challenges because sometimes they are made pregnant by men that have not married them, men that are not committed to marrying them, and then some parents are usually vey harsh. In that mix up; when you get pregnant and the parent had paid your school fees, and there is nothing else you can say to her. So this girl simply sneaks away and terminates her life!

**M: Everyone will participate in answering this one;**

R: (light chuckles)

**M: I had asked; what reasons do you have in your community that contributes to the young teenage girls contemplating terminating their lives when they get pregnant?**

R: No. 6 – You might decide to terminate your life by; for instance if you are a young teenage girl, you may get pregnant. When you get pregnant, if you get pregnant and your parents come to learn of it, and its not like they were not supportive; they have paid your school fees and the find you been disobedient in all they tell you, and perhaps they keep on abusing you to the extend that you cannot find peace. They abuse you; girls in most cases are abused together with their mothers! Or directing the abusive words to the Mothers’ character! You are told ‘go, go, your mother has taught you that’ and so you have no peace with the parents. You disagree with the parents to the extend that you cannot know peace and so instead of always having conflicts with the parent you choose / and it’s not like you are saying…. Let’s say for instance; someone has made you pregnant and they had no plans of marrying you, and here you are; you are leaving your home where you were born and you are being chased away! So you decide; instead of always being abused; you choose to consider terminating your life. You find that you are having a lot of stress to terminate your life.

**M: Ok, what other reasons?**

R: Silence

**M: Here, here, here, please pick it and say something! It will not bite you! Number, and then simply say your number?**

R: Silence.

**M: Say something. What is your number? Number seven?**

R: Silence

**M: In the community, do you hear of young teenage girls or persons that have been contemplating terminating their lives? Do you have that problem?**

R: Silence

**M: Do you have that problem?**

R: Silence

**M: Yes,**

R: No. – Yes, those problems are there especially when children; young children like these young girls when they are orphaned by their parents. In many cases they are adopted by church sponsors or certain parents who go and educate them. Now when this girl gets pregnant, she feels guilty going back to her sponsors to inform them because they will get de motivated to support her. So she decides to terminate her life.

**M: Had you said your number?**

R: No. 5

**M: Yes, who else? We were asking about the reasons. The reasons that make young teenage girls want to terminate their lives when they fall pregnant?**

R: Silence

**M: Number three have you said anything?**

R: No. 3 – No.

**M: And you have not heard?**

R: No. 3 – Sorry?

**M: And you have not heard? You have not heard?**

R: No. 3 - No

**M: Silence**

R: No. 3 – In my community I have not heard of any.

**M: Who?**

R: No. 3 – Who had contemplated to terminate her life.

**M: Ok, lets wide up. We have said when you are having mental disorders; and especially stress, whom do you prefer knowing first? Whom do you prefer to inform first? Is it your mother, your husband or your TBA? Whom do you prefer reaching to first for support?**

R: No. 4 - My husband.

**M: Number?**

R: No. 4

**M: Yes,**

R: No. 5 – My husband and parents

**M: Yes,**

R: No. 6 – My parents.

**M: Is there anything else you would wish to share? As we finish our discussion?**

R: Silence

**M: We want to finish our discussions. Anything else?**

R: Silence

**M: Ok, we are so grateful, yes.**

************* End *************
